# Treatment of Amebic Dysentery with Ipecac by the Rectum

**Published:** 1919

**Authors:** 


***The Treatment of Amebic Dysentery with Ipecac by the Rectum.*** By G. B. Lawson. *The Journal of the American Medical Association*, September 28, 1918.

The author describes a method of treatment of amebic dysentery, similar to that of Brem and Zeiler. This method consists in putting from 60 to 120 grains of powdered ipecac into about 24 ounces of water; this is kept hot for an hour, but not allowed to boil. After washing out the bowel with warm water, the whole preparation is given slowly by rectum to be retained as long as possible. In the majority of cases, salol-coated ipecac pills are given at the same time, or emetin used hypodermically.

Dr. Lawson believes that the hypodermic emetin treatment for dysentery can be greatly aided by also giving ipecac by the rectum, and that it is better to use ipecac by rectum than hypodermically where it will diffuse through all the tissues of the body by the time it reaches the seat of the disease. The administration of ipecac by rectum has also the advantage that it can be used by the patient when away from the physician.

